# Deep learning-enhanced digital-BGO versus TOF PET/CT: comparative assessment of detection, quantitation, and overall image quality

**DOI:** 10.1186/s40658-025-00814-8

**Published:** 2025-12-16

**Authors:** Quentin Maronnier, Thibaut Cassou-Mounat, Erwan Gabiache, Adrien Latgé, Marie Terroir, Lavinia Vija, Kuan-Hao Su, Olivier Caselles, Frédéric Courbon

**Affiliations:** 1 Medical Physics Department, Oncopole Claudius Regaud, Toulouse, France; 2 Nuclear Medicine Department, Oncopole Claudius Regaud, Toulouse, France; 3 GE HealthCare, Chicago, IL USA

**Keywords:** Positron-emission tomography, Deep learning, Detection, Quantitation, Image quality, Simulation

## Abstract

**Background:**

We evaluate the Omni Legend 32 cm (OMNI6R), a digital-BGO PET/CT using the deep learning (DL) based algorithm, Precision Deep Learning (PDL), emulating time-of-flight (TOF) enhancement and compare its performance to the TOF-equipped Discovery-MI 25 cm (DMI5R) in terms of detection sensitivity, quantification, and overall image quality.

**Methods:**

Thirty patients were administered with an average single dose of 2 MBq/kg [^18^F]-FDG and were scanned consecutively on DMI5R first and on OMNI6R afterwards. Total scan duration on DMI5R and OMNI6R were 10 and 6 min, respectively. OMNI6R data were reconstructed using Bayesian Penalized Likelihood (BPL) algorithm with a beta of 650 and PDL-High setting. A total of 150 inserted synthetic lesions (ISL), ranging in size from 6 to 10 mm and exhibiting contrast levels between 3 and 15 relative to their initial background activity, were distributed across the cohort. Three readers blindly assessed detection sensitivity and quantification of these lesions. We tested a non-inferiority hypothesis based on the ISL true positive rate (TPR) and compared calculated recovery coefficients (RC) using SUVmean and SUVmax metrics of the detected ISL. Additionally, image quality, sharpness, conspicuity, noise characteristics, and diagnostic confidence were assessed as clinical quality indicators with a 5-point Likert scale on clinical images without ISL, using same beta as DMI5R and different PDL settings (None, High, Medium, Low).

**Results:**

TPR were 84.67% (95% CI 80.04–89.29%) and 84.44% (95% CI 77.76–91.13%) respectively for DMI5R and OMNI6R-PDL-High, and demonstrated non-inferiority. OMNI6R-PDL-High yielded higher RC without overestimation for all ISL sizes. Remarkably, these findings were observed despite a 9% activity decay in ISL and a 40% reduction in whole-body acquisition time. All PDL settings led to increased average median scores across clinical quality metrics, surpassing the DMI5R in most cases.

**Conclusions:**

OMNI6R using PDL-High demonstrated non-inferior diagnostic performance compared to DMI5R, as evidenced by ISL detection sensitivity and quantitation. Importantly, the use of OMNI-PDL-High did not increase the risk of false-negative findings, despite reductions in activity and acquisition time. OMNI6R using PDL enhances overall image quality while improving clinical workflow and patient comfort. These results support DL-based enhancement algorithms as effective solutions for non-TOF PET imaging.

*Trial registration number and date of registration:* NCT05154877, December 13th 2021.

## Background

Positron emission tomography combined with computed tomography (PET/CT) is being extensively employed in various oncological contexts, Including diagnosis, staging and surveillance [1].

When a new PET/CT technology is introduced, it is crucial to provide clinicians with a comprehensive performance evaluation to ensure accurate system characterization. A key challenge in PET imaging is minimizing scan time to enhance patient’s comfort and workflow while simultaneously maximizing sensitivity, with the aim of improving detection and quantification of subtle pathological uptakes [2, 3]. To improve the performance of current PET systems, ongoing development is focusing on deep learning (DL) based enhancement algorithms [4]. Such innovations must be rigorously assessed to optimize diagnostic accuracy by mitigating the risk of false positive and false negative rates [5]. Nonetheless, as the real-world clinical settings lack of a definitive ground truth reference, caution is needed when claiming performance gains in this context. Moreover, integrating deep learning into the software architecture of PET systems adds a further layer of complexity.

The Omni Legend 32 cm (OMNI6R; General Electric Healthcare, Chicago, IL, USA), a PET/CT system equipped with bismuth germanium oxide (BGO) crystals coupled with Silicon Photomultiplier (SiPM) technology was installed in our institution Institut Universitaire du Cancer Toulouse Oncopole (IUCT-O; Toulouse, France) in 2022, as an experimental system for its physical [6, 7] and clinical assessments. However, as lacking sufficient Time-of-Flight (TOF) timing resolution for image reconstruction, OMNI6R is designed to emulate the TOF effect on the images using a DL-based PET enhancement algorithm named “Precision Deep Learning” (PDL), intended to improve the signal-to-noise ratio (SNR) and enhance the detection and quantitation of small lesions [8].

The inserted synthetic lesion (ISL) method is a simulation strategy which modifies the clinical data for creating a controlled ground-truth reference within a realistic anatomical model, allowing the evaluation and comparison of PET/CT scanners in clinical settings. The ISL method is particularly valuable for evaluating DL-based reconstruction algorithms, as it directly assesses their impact on clinical data. The feasibility and accuracy of the ISL method was previously demonstrated in an experimental controlled setup [9], and its clinical relevance was comparatively validated on different PET/CT systems [10].

Considering that DL-based algorithms like PDL might have an impact on detection, quantification, and overall image quality in PET imaging [4], its careful validation seems required. The present study aimed to test OMNI6R with PDL against a TOF PET/CT system, on the one hand, using ISL as reference for assessing detection and quantification and, on the other hand, examining the qualitative impact of various PDL settings on OMNI6R. We hypothesize that OMNI6R performs comparably to standard TOF PET/CT systems while containing acquisition times.

## Materials and methods

### Study design and participant

A monocentric, prospective paired clinical trial (NCT05154877) was conducted at the Institut Universitaire du Cancer Toulouse–Oncopole (IUCT-O; Toulouse, France) and sponsored by the manufacturer (General Electric Healthcare, Chicago, IL, USA). First, our primary objective of this study was to verify the non-inferiority of the OMNI6R system using PDL in terms of ISL detection as compared to a TOF PET/CT, the Discovery-MI 25 cm (DMI5R). The sample size was calculated based on a lesion-level analysis [11, 12]. A target of 30 subjects, each with an average of 5 ISL, was projected to provide at least 80% power to detect non-inferiority using McNemar’s test for paired binary outcomes. Second, we assessed the quantification of the detected ISL for each PET/CT system. Additionally, we qualitatively evaluated the clinical datasets generated by OMNI6R for different PDL settings along with those by DMI5R without using ISL.

All patients included underwent two consecutive PET/CT scans using DMI5R and OMNI6R scanners. They had an oncologic indication for [^18^F]-FDG PET/CT based to local practice standards, were over 18 years old with an Eastern Cooperative Oncology Group (ECOG) performance status ≤ 1 and a Karnofsky index > 70, and were able to maintain a strict supine position during both scans. Key exclusion criteria encompassed uncompensated diabetes and formal contraindications to certain imaging procedures (severe claustrophobia, heart valve issues, pacemaker, etc.). All patients provided written informed consent. In this study, a dataset refers to a paired scan-rescan acquisition for a given subject, meaning that each dataset comprises two scans of the same patient performed consecutively on the two different PET systems.

### PET/CT systems

Of the PET/CT scanners employed in this study, DMI5R features a five-ring configuration, providing an axial Field-Of-View (FOV) of 25 cm, and the properties of its lutetium yttrium oxyorthosilicate (LYSO) crystals natively enables TOF reconstruction. By contrast, OMNI6R has a six-ring configuration, which offers a 32-cm axial FOV, and integrates a deep-learning algorithm (PDL) emulating the TOF effect, deemed capable to improve the SNR and to enhance the detection and quantitation of small lesions from non-TOF acquisitions [7, 13]. PDL is executed following reconstruction via Q.Clear (GE Healthcare, Chicago, IL, USA), which is based on the Bayesian penalized-likelihood (BPL) algorithm [14]. Three enhancement models are available: High, Medium, and Low. The parameters have different levels of contrast-enhancement-to-noise trade-off to reflect the target level of contrast-to-noise [4]. For example, PDL-High (PDL-H) greatly increases contrast while having a minimal effect on noise.

### Acquisition and reconstruction

Patients were injected with an average single dose of 2 MBq/kg of [^18^F]-FDG. Consecutive acquisitions were performed at a median uptake time of 61 min and 79 min for DMI5R and OMNI6R respectively. Each scanning session started with the DMI5R—i.e. the standard of care—followed by OMNI6R.

The first CT acquisition performed on DMI5R followed clinical practice, using a modulated high tube voltage as well as an adjusted noise index based on each patient’s shape and body mass index. The second CT acquisition performed on OMNI6R followed the same protocol although adjusted for minimizing radiation exposure to the levels of low-dose CT.

For PET acquisitions performed on the DMI5R system, we used standard clinical protocols. Scans covered five bed positions with a 25% overlap and an acquisition time of 2 min per FOV, resulting in a total scan duration of 10 min. The transverse FOV was adapted to each patient’s anatomy, with values of 500, 600, or 700 mm. A predefined beta value was applied accordingly: 450, 500, or 550, respectively. Regarding the OMNI6R, leveraging the extended axial FOV of 32 cm and a reduced acquisition time of 1.5 min per bed position (with the same 25% overlap), the total scan duration was shortened to 6 min. For the reconstruction performed on the OMNI6R, two distinct approaches were applied, corresponding to the two parts of the study. For the detection and quantification analysis, the primary focus of the study, an optimized protocol was designed based on prior experimental and retrospective clinical studies. A fixed beta value of 650 coupled with PDL-H was applied across all patients to ensure consistency in quantification. The transverse FOV was adapted to match the value used on the DMI5R for each individual. These acquisition and reconstruction parameters were optimized in accordance with the latest EANM Research Ltd. (EARL) guidelines [15, 16], allowing a 40% reduction in acquisition time compared to standard clinical protocols.

For the qualitative analysis, the objective was to compare the impact of different PDL settings on the overall image quality, in relation to both DMI5R and native OMNI6R images. To isolate the effect of PDL, the reconstruction parameters used during the initial DMI5R acquisition, specifically the transverse FOV and beta value, were replicated. This approach, conducted prior to the implementation of the optimized protocol for the detection and quantification study, was intended to assess the influence of varying PDL settings on visual overall image quality.

PET raw data were reconstructed into images using BPL with and without PDL for OMNI6R and with TOF for DMI5R. PET acquisition and reconstruction parameters are detailed in Table 1. PET and CT data were anonymized, transferred and stored to a research workstation.

**Table 1:** PET acquisition and reconstruction parameters used with DMI5R and OMNI6R systems

	DMI5R	OMNI6R
Radiotracer	[^18^F]-FDG	
Posology (MBq/kg)	2	
Average uptake time (min)–Median [min; max]	61 [57; 70]	79 [73; 93]
Axial FOV (cm)	25	32
Overlap (%)	25	25
Time per FOV (min/FOV)	2	1.5
Total acquisition time (min)	10	6
Matrix size	256 × 256	384 × 384
Transverse FOV (mm)	[500, 600, 700]	[500, 600, 700]
Reconstruction algorithm	BPL	
Beta–detection & quantification evaluation using ISL	[450, 500, 550]	650
Beta–overall image quality evaluation	[450, 500, 550]	[450, 500, 550]
Corrections	Attenuation, random, scatter, point spread function	
TOF	Yes	No
PDL	No	Yes

### Detection and quantification evaluation using ISL

A detection and quantification assessment was performed on two datasets: the DMI5R and the OMNI6R using PDL-High (OMNI6R-PDL-H). One setting of PDL was selected to ensure statistical significance in the non-inferiority study, opting for PDL-H as it has the most significant impact on contrast and therefore on detection and quantification [4].

Based on previously described [10], synthetic lesions were inserted on PET data from two consecutives exams at the same anatomical location for each patient. We determined the same anatomical location from the CT images and measured the semi-quantitative mean Standardized Uptake Value (SUVmean) (g/L) using a 2 cm^3^ spherical volume-of-interest (VOI) prior to the simulation for each PET scans. It was done to ensure consistent level of activity considering the same anatomical location on consecutive exams of the same patient. The average SUVmean measured at the ISL locations was, on average, 9% lower on the OMNI6R images compared to the DMI5R ones, due to radioactive decay. The modelling, simulation and reconstruction were performed on a dedicated Z8 workstation (Hewlett-Packard, Palo Alto, CA, USA) using the research toolbox Duetto (v02.18, General Electric Healthcare, Chicago, IL, USA) and executed with MATLAB R2018b (The MathWorks Inc., Natick, MA, USA).

A total of 150 synthetic lesions were dispatched among the 30 enrolled patients. These lesions were homogeneous and spherical, with diameters of 6, 8, and 10 mm, and contrast ratios relative to their background ranged from 3 to 15. To facilitate the distribution of tumor burden, the cohort was divided into three groups of 10 patients each: group M0, with no ISL; group OligoM, with 1 to 5 ISL per patient (3 ISL on average); group MultiM, with 10–15 ISL per patient (12 ISL on average). The size and contrast of the synthetic lesions were not evenly distributed across the cohort either. Synthetic lesions were distributed across consecutive scans at consistent anatomical locations. Table 2 shows the characteristics of synthetic targets in terms of shape, diameter, contrast and anatomical location.

**Table 2:** Description of synthetic lesions

Number	150
Shape	Spherical
Diameter (mm) [min; max]	6–10
Contrast [min; max]	3–15
*Anatomical location–Number (proportion (%))*	
Head and neck	25 (16.7%)
Other lymph nodes	16 (10.7%)
Mediastinum	26 (17.3%)
Lungs	22 (14.7%)
Liver	33 (22.0%)
Bone	28 (18.7%)
Abdomen	25 (16.7%)

**Fig. 1:**
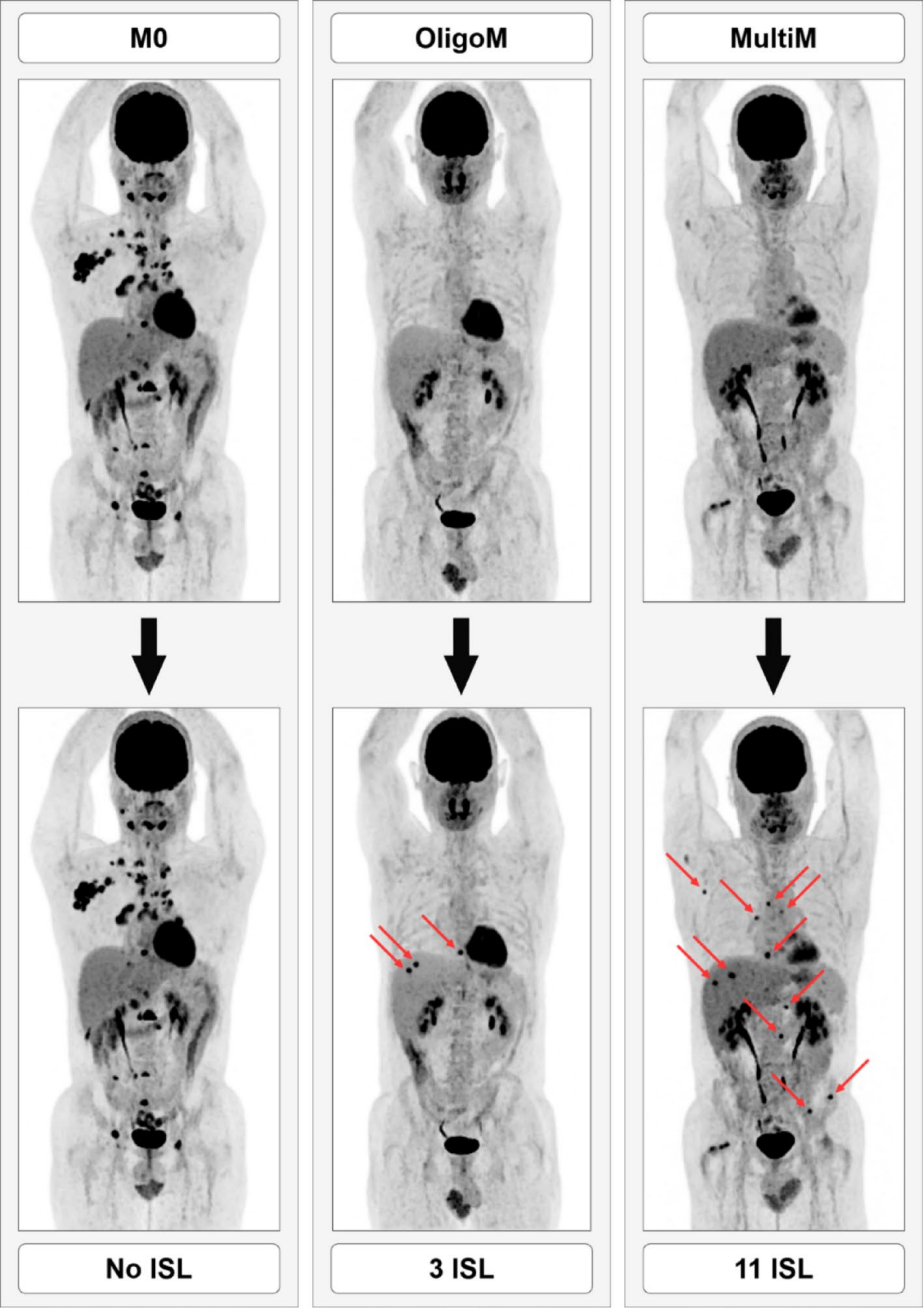
Representative patient template showing lesion burden variation for M0, OligoM, and MultiM groups. A case from the M0 group with no ISL (left), an OligoM case with 3 ISL (center), and a MultiM case with 11 ISL (right). Red arrows indicate ISLs distributed across multiple anatomical regions. All cases displayed were acquired on the OMNI6R system using a fixed beta value of 650 and the PDL-H setting

Figure 1 presents representative cases from each group, illustrating the distribution across the study population.

Since the PDL was not available directly on Duetto, the industrial partner applied it remotely to the anonymized images modified by the OMNI6R simulation and returned them. We processed the DMI5R images using our available tools. All the images were imported and stored on the AWServer client console (General Electric Healthcare, Chicago, IL, USA) for the reading sessions using the image interpretation protocol Volume Viewer (PETVCAR®). Three nuclear medicine physicians independently evaluated the images in blinded conditions by reporting detected lesions and measuring the SUVmean and SUVmax. The datasets were evaluated in random order, with observers blinded to both the PET system used and the number and location of ISL.

### Overall image quality evaluation

We used three datasets: one from the first scanner acquisition (DMI5R), another from the second scanner acquisition (OMNI6R), and a third from the second acquisition using PDL (OMNI6R-PDL). Various PDL settings were applied to assess their impact on qualitative metrics in comparison to DMI5R and native OMNI6R. Out of the 30 scans in the OMNI6R-PDL dataset, there were 12 PDL-High, 13 PDL-Medium, and 5 PDL-Low scans.

**Fig. 2:**
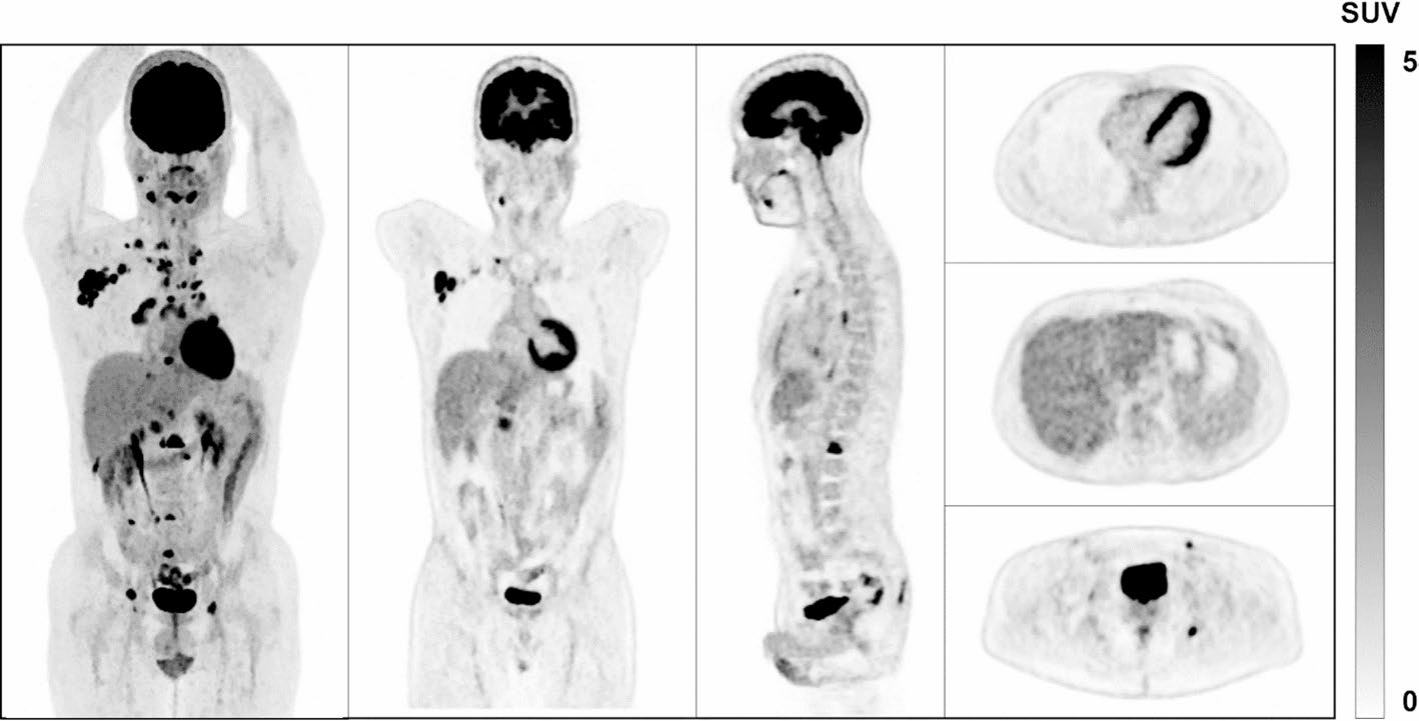
Example of static templates from the overall image quality assessment. From left to right: the MIP visualization, the coronal slice, the sagittal slice, and the three transaxial slices. Images were acquired on the OMNI6R system using a beta value of 500 and the PDL-H setting

A review template was developed to visualize anatomical structures of interest, facilitating a discriminative and quick assessment of the overall quality of the scans. The templates were created using a black-and-white inverse linear scale. They included an anterior view of the maximum intensity projection (MIP), a coronal slice through the mediastinum and liver region, a sagittal slice capturing the spine and the aortic cross, and three transaxial slices covering the lung and heart, liver, and bladder to represent various intensity levels. Three nuclear medicine physicians independently and blindly evaluated each template using a 5-point Likert scale to score four quality indexes: image quality (IQ), sharpness and conspicuity (SC), noise characteristics (NC), and diagnostic confidence (DC). The 5-point scale was defined as follows: 5–Diagnostic, Excellent; 4–Diagnostic, Good; 3–Acceptable; 2–Sub-optimal Diagnostic; 1–Non-Diagnostic. The templates were assessed in random order, and the observers were unaware of which dataset was displayed. Figure 2 presents a review template created.

### Statistical analysis

For detection sensitivity, we assessed the non-inferiority of the OMNI6R-PDL-H system compared to the DMI5R PET/CT in terms of ISL true positive rate (TPR). TPR were calculated as the ratio of detected ISL to the total number of ISL. The non-inferiority of OMNI6R-PDL-H was evaluated against the DMI5R with a margin of − 7%. For quantitation, we calculated the average recovery coefficient for SUVmean (RC-SUVmean) and SUVmax (RC-SUVmax) for ISL by dividing the measured SUVmean and SUVmax by the simulated value. For the overall image quality assessment, we calculated the mean of the median scores for each dataset overall, and then separated the results according to the PDL settings. A Wilcoxon signed-rank test was applied to the overall dataset to compare the median scores of OMNI6R-PDL with those of OMNI6R and DMI5R for each quality index. A *p* value of < 0.05 was considered statistically significant. Uncertainty was expressed via 95% confidence interval (CI).

## Results

A total of 30 patients were enrolled in this prospective monocentric study between the 1st of June and the 30th of August 2022, with the following characteristics recorded at inclusion (median [min; max]): age (years): 61.5 [28; 83]; weight (kg): 73.5 [47; 98]; height (cm): 168 [156; 184]; and body mass index (BMI, kg/m^2^): 24.9 [18.8; 35.3].

### Detection and quantification evaluation using ISL

Overall, of the 450 potentially detectable synthetic lesions, the readers detected 381 and 380 respectively on DMI5R and OMNI6R-PDL-H, while 346 and 356 natural lesions were respectively identified therein. Reader-specific results are detailed in Table 3. The TPR for DMI5R and OMNI6R-PDL-H was 84.67% and 84.44%, respectively. The ISL TPR difference between DMI5R and OMNI6R-PDL-H (σ_DMI5R_–_OMNI6R-PDL-H_) was − 0.22%, with a 95% CI ranging from − 5.19 to 4.75%. Since the lower bound (− 5.19%) exceeds the predefined non-inferiority margin of –7%, non-inferiority is demonstrated (Table 4).

**Table 3:** Number of synthetic and natural lesions detected for DMI5R and OMNI6R-PDL-H by each reader

	Number of synthetic lesions detected		Number of natural lesions detected	
	DMI5R	OMNI6R-PDL-H	DMI5R	OMNI6R-PDL-H
Reader 1	130	134	120	126
Reader 2	123	123	112	110
Reader 3	128	123	114	120
Total	381	380	346	356

**Table 4:** TPR of synthetic lesions according to the PET system

Modality	TPR of synthetic lesions		
	Estimate (%)	95% CI	
		Lower limit	Upper limit
DMI5R	84.67	80.04	89.29
OMNI6R-PDL-H	84.44	77.76	91.13
σ_DMI5R – OMNI6R-PDL-H_	− 0.22	− 5.19	4.75

Averaged across all readers, TPR by lesion size (6-, 8-, and 10-mm) were 91.7%, 90.0%, and 69.8% with the DMI5R, and 95.2%, 82.9%, and 80.2% with the OMNI6R, respectively.

In terms of anatomical location, detection performance varied depending on the region. The hepatic region consistently showed the highest detection rates across both systems. Pulmonary and bone lesions were more frequently detected with OMNI6R-PDL-H compared to DMI5R, whereas DMI5R demonstrated better detectability for synthetic lesions located in the head and neck, mediastinal, lymph node, and abdominal regions.

**Fig. 3:**
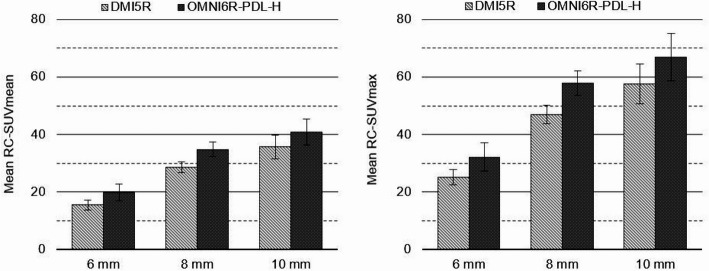
Mean RC-SUVmean (left panel) and RC-SUVmax (right panel) for DMI5R and OMNI6R-PDL-H in clustered bar plots. The error bars represent the 95% CIs

Considering the ISLs detected on DMI5R, the mean RC-SUVmean was 15.4%, 28.5%, and 35.7% for 6-, 8-, and 10-mm lesions, respectively, while the corresponding values for OMNI6R-PDL-H were 19.9%, 34.8%, and 40.9%. Moreover, the mean RC-SUVmax for DMI5R were 25.2%, 46.9%, and 57.6% for 6-, 8- and 10-mm lesions, respectively, while the corresponding values for OMNI6R-PDL-H were 32.2%, 57.8%, and 66.9% (Fig. 3).

### Overall image quality evaluation

**Fig. 4:**
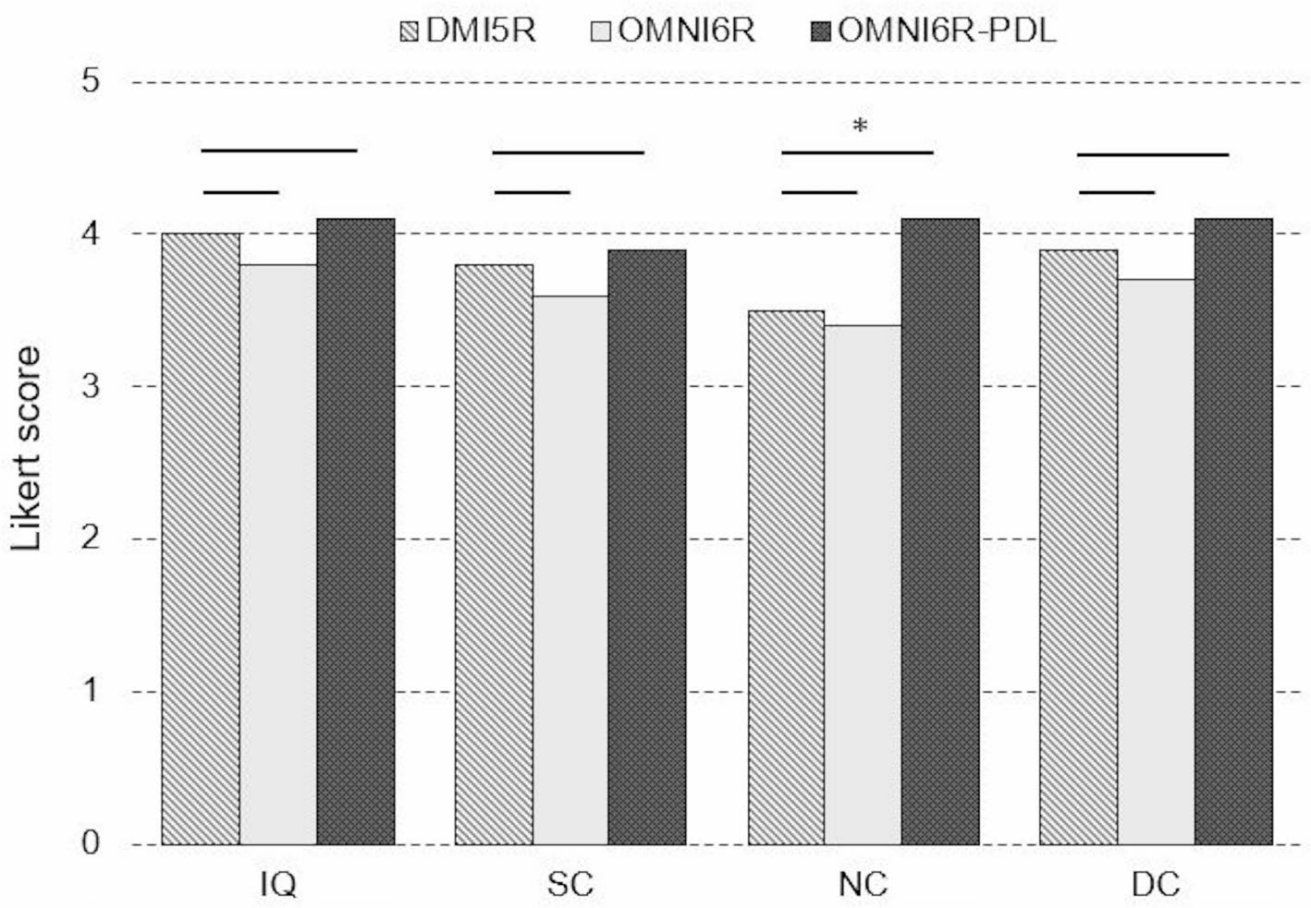
Mean of median quality scores for each dataset (DMI5R, OMNI6R, and OMNI6R-PDL) in clustered bar plots. A consistent beta value (450, 500 or 550) was applied across all three systems within each dataset, with the beta value varying only between datasets. The Wilcoxon signed-rank test results are indicated by the presence or absence of asterisks above the lines linking the datasets. One asterisks indicate a significant difference at *p* < 0.05

The mean of median scores for IQ, SC, NC, and DC were as follows: 4.0, 3.8, 3.5, and 3.9 for the DMI5R; 3.8, 3.6, 3.4, and 3.7 for the OMNI6R; and 4.1, 3.9, 4.1, and 4.1 for the OMNI6R-PDL. The only statistically significant difference was detected for NC (*p* = 0.0003) (Fig. 4).

**Fig. 5:**
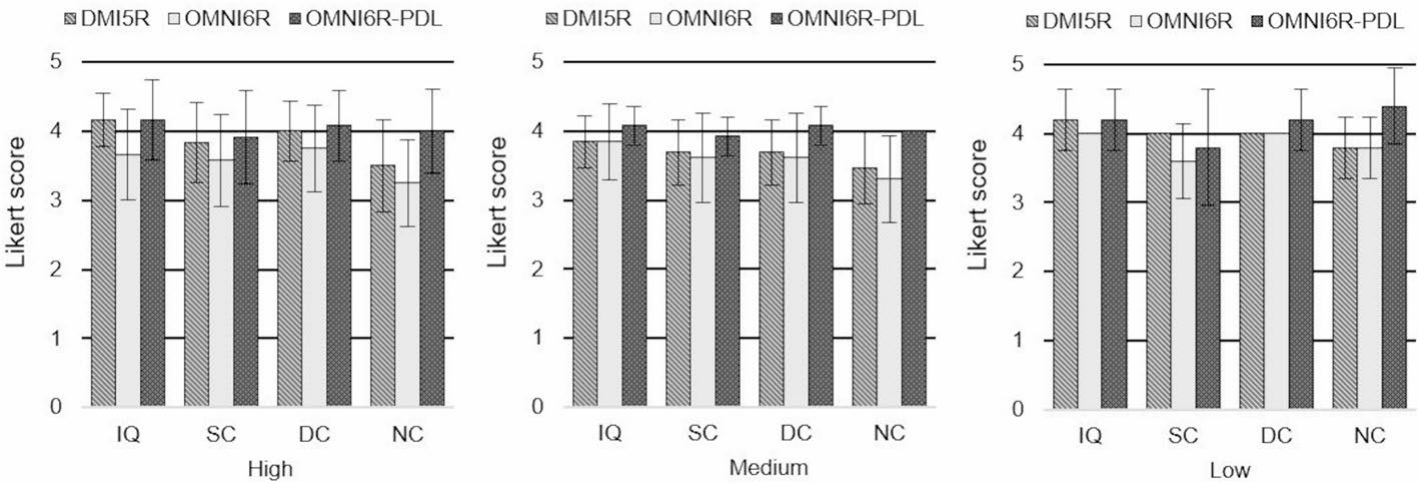
Mean of median quality scores for each dataset (DMI5R, OMNI6R, and OMNI6R-PDL) and PDL settings in clustered bar plots. From left to right: PDL-High (left panel, n = 12), PDL-Medium (center panel, n = 13), and PDL-Low (right panel, n = 5). A consistent beta value (450, 500, or 550) was applied across all three systems within each dataset, with the beta value varying only between datasets. Error bars represent the standard deviations of measurements within each group

The results obtained for the different PDL settings separately are presented in Fig. 5. The patient populations shown in each plot are distinct and correspond to different datasets.

## Discussion

Introducing new technologies in clinical practice, especially those featuring DL, requires thorough evaluation in not only experimental but also real-world clinical settings [17]. While visual comparison is appropriate for an initial assessment, it remains insufficient for assessing the diagnostic performance particularly the detection sensitivity and quantification accuracy related to these new technologies. A key challenge in evaluating new PET technologies is the lack of reliable clinical ground-truth techniques, hampering performance assessment on patient data. To address this, the use of ISL data offers a controlled and realistic approach to quantify the impact of these innovations [18], and its combination with the scan-rescan methodology enables performance comparison across different systems [10]. All patients underwent sequential imaging, with the DMI5R scan performed first for clinical purposes, followed by the OMNI6R scan, which was conducted exclusively for research. This fixed order was necessary due to clinical workflow constraints and regulatory limitations on the research system. However, such sequencing introduces potential biases: tracer decay leads to reduced activity during the second scan, while prolonged uptake time may enhance lesion-to-background contrast, potentially benefiting the latter acquisition [19]. To minimize these biases, we focused the detection and quantification study on ISL, allowing precise control over lesion size and contrast. This approach helped mitigate the inherent limitations of the study, ensuring a more reliable assessment of both systems. It supports a rigorous and clinically relevant evaluation of new PET/CT technologies—particularly important in the context of DL-based algorithms, which may introduce artifacts or lead to overdiagnosis [20, 21].

In this study, we comparatively evaluated a digital-BGO PET/CT system with DL-based image enhancement (OMNI6R) against a system natively capable of TOF reconstruction (DMI5R). PDL trained using Discovery-MI models, serves as an appropriate candidate for performance comparison [4]. Our primary objective is to assess the non-inferiority of the OMNI6R scanner compared to the DMI5R using synthetic lesions to simulate consistent pathological uptake across consecutive PET acquisitions, enabling calculation of the true positive rate (TPR). For detection and quantification evaluation, we selected a single PDL setting to ensure statistical significance in the non-inferiority study. We chose the PDL-High since it has the greatest impact on PET quantification [4], making it the most suitable choice for detecting any potential overestimation. The sizes and contrasts of the ISLs are intended to evaluate the systems’ capabilities within current detection and quantification limits of the PET systems technology [22]. For OMNI6R, an optimized protocol with a fixed beta value of 650 and PDL-H was applied. Semi-quantitative comparisons were then performed by evaluating the SUV measurements of detected ISLs across both systems. Additionally, a qualitative assessment was conducted using native scans without ISLs to compare overall image quality and evaluate the influence of different PDL settings. Reconstruction parameters from the DMI5R protocol were replicated, with the exception of an increased matrix size. This comprehensive and innovative approach integrates detectability, semi-quantitative accuracy with ground truth, and qualitative evaluation from a reader’s perspective.

We aimed to determine whether OMNI6R-PDL-High performs at least as well as DMI5R in lesion detection, using a non-inferiority margin of 7%. Non-inferiority was demonstrated, as the lower bound of the 95% confidence interval for the difference between DMI5R and OMNI6R-PDL-High remained above the predefined margin. The detection rate of around 84% for synthetic lesions is reasonably satisfactory given their size and activity, and reflects the detection limits of current PET systems. This contrasts with previous studies showing clearer performance gaps, where differences were easier to detect [10].

In terms of lesion detection, as expected, TPR decreased with smaller lesion sizes, highlighting the inherent limitations of PET systems in reliably identifying sub-centimeter lesions. While the OMNI6R demonstrated higher detection rates for the smallest lesions, the DMI5R performed slightly better for the 8 mm lesions. But, no significant trend favouring one system over the other was observed across lesion sizes. Regarding anatomical location, we draw the same conclusion: although regional differences in detection performance were observed between the two systems, no definitive conclusions can be made about their relative effectiveness across different anatomical sites.

The semi-quantitative analysis showed slightly improved quantification accuracy with OMNI6R-PDL-H compared to DMI5R, as highlighted by higher mean RC-SUVmean and RC-SUVmax values across all lesion sizes, without any signs of overestimation. Despite a reduced scan time and lower activity concentration within the ISLs, the lesion detection and quantification performance of OMNI6R-PDL-H remained non-inferior. The reduction in the acquisition time is a result of the optimization of OMNI6R acquisition protocols, whereas the decrease in activity stems from the physical and pharmacokinetics properties of the radiotracer. Based on existing research on PDL, we hypothesize that other parameters, with their lesser effect on quantification, should not lead to a systematic overestimation of lesion uptake [4, 17, 21]. However, we did not compare the impact of different PDL settings on detection and quantification, which we expect to decrease depending on the chosen setting.

Regarding the overall image quality assessment, PDL enhances every quality indexes, as evidenced by the mean median scores. While OMNI6R without PDL shows slightly lower values compared to DMI5R, the application of PDL improves all evaluated criteria, even surpassing DMI5R scores in most cases. The Wilcoxon test performed on the overall datasets revealed a significant difference in NC, with a higher score for OMNI6R-PDL compared to DMI5R. This improvement likely results from the combination of the high sensitivity brought by the digital detectors coupled with the noise-reducing capabilities of PDL [4, 7]. When evaluating the effect of each PDL setting, we observed a global increase in scores across all quality metrics. For PDL-H and PDL-M, the scores were consistently higher than those of OMNI6R and were either higher or equivalent to those of DMI5R. For PDL-L, while scores remained higher than OMNI6R, only the NC and DC metrics exceeded the TOF system scores. The IQ metric was equivalent to those of the TOF system, while SC showed lower score. This finding is likely due to the more pronounced smoothing effect from PDL-L, which does not significantly improve delineation of organs in the images [21]. Our findings are consistent with other studies evaluating the qualitative impact of PDL on clinical data [21]. In this study, we focused on comparing the quality metrics between DMI5R and OMNI6R with and without PDL, as we did not apply each PDL parameter to every patient.

These results highlight the benefit of such technology implementation to ensure robust clinical performance through lesion detection, quantification, and overall image quality even under challenging conditions (i.e. reduced acquisition time and activity). Such enhancement algorithm could be advantageous compared to TOF systems in terms of production cost, especially if the performance gains offered are comparable to the improvements associated with TOF [23].

We can highlight a limitation regarding the realism of ISL. Incorporating synthetic lesions with irregular shapes and heterogeneous uptake could help address this limitation, enhance realism, and allow for a more in-depth performance analysis.

## Conclusion

Through a scan-rescan approach using ISLs as a controlled ground-truth reference, the OMNI6R system with PDL-High demonstrated non-inferior detection sensitivity and quantification accuracy compared to DMI5R, despite lower activity in ISL and reduction in acquisition time. Overall image quality assessments further revealed that PDL globally enhanced clinical metrics, mostly surpassing the TOF-equipped system. Integrating DL-based enhancement algorithms into non-TOF PET/CT systems improve diagnostic performance, while improving workflow efficiency, reducing patient discomfort, and offering a cost-effective alternative to TOF systems. Our findings support the integration of DL-based algorithms like PDL as a promising solution for non-TOF PET scanners, highlighting the potential for the widespread implementation of this technology in routine clinical practice. Further studies seem warranted to assess its performance across diverse clinical settings, including different radiotracers and acquisition protocols.

## Data Availability

The datasets used and/or analysed during the current study are available from the corresponding author on reasonable request.

## Abbreviations

PET: Positron emission tomography
CT: Computed tomography
DL: Deep learning
OMNI6R: Omni legend 32 cm PET/CT
BGO: Bismuth germanium oxide
SiPM: Silicon photomultiplier
TOF: Time-of-flight
PDL: Precision deep learning
SNR: Signal-to-noise ratio
ISL: Inserted synthetic lesion
DMI5R: Discovery-MI 25 cm PET/CT
ECOG: Eastern Cooperative Oncology Group
FOV: Field of view
LYSO: Lutetium–yttrium oxyorthosilicate
BPL: Bayesian penalized likelihood
EANM: European Association of Nuclear Medicine
EARL: EANM Research Ltd
SUV: Standardized uptake value
VOI: Volume-of-interest
MIP: Maximum intensity projection
IQ: Image quality
SC: Sharpness and conspicuity
NC: Noise characteristic
DC: Diagnostic confidence
TPR: True positive rate
RC: Recovery coefficient
CI: Confidence interval
BMI: Body mass index
